# A Novel Electrochemical Sensor Based on Pd Confined Mesoporous Carbon Hollow Nanospheres for the Sensitive Detection of Ascorbic Acid, Dopamine, and Uric Acid

**DOI:** 10.3390/molecules29112427

**Published:** 2024-05-21

**Authors:** Wanqing Zhang, Xijiao Li, Xiaoxue Liu, Kaixuan Song, Haiyang Wang, Jichao Wang, Renlong Li, Shanqin Liu, Zhikun Peng

**Affiliations:** 1 School of Chemistry and Chemical Engineering, Henan Institute of Science and Technology, Xinxiang 453003, Chinawangjichao@hist.edu.cn (J.W.);; 2 China Henan Institute of Advanced Technology, Zhengzhou University, Zhengzhou 450001, China

**Keywords:** electrochemical sensor, Pd/MCHS, ascorbic acid, dopamine, and uric acid

## Abstract

In this study, we designed a novel electrochemical sensor by modifying a glass carbon electrode (GCE) with Pd confined mesoporous carbon hollow nanospheres (Pd/MCHS) for the simultaneous detection of ascorbic acid (AA), dopamine (DA), and uric acid (UA). The structure and morphological characteristics of the Pd/MCHS nanocomposite and the Pd/MCHS/GCE sensor are comprehensively examined using SEM, TEM, XRD and EDX. The electrochemical properties of the prepared sensor are investigated through CV and DPV, which reveal three resolved oxidation peaks for AA, DA, and UA, thereby verifying the simultaneous detection of the three analytes. Benefiting from its tailorable properties, the Pd/MCHS nanocomposite provides a large surface area, rapid electron transfer ability, good catalytic activity, and high conductivity with good electrochemical behavior for the determination of AA, DA, and UA. Under optimized conditions, the Pd/MCHS/GCE sensor exhibited a linear response in the concentration ranges of 300–9000, 2–50, and 20–500 µM for AA, DA, and UA, respectively. The corresponding limit of detection (LOD) values were determined to be 51.03, 0.14, and 4.96 µM, respectively. Moreover, the Pd/MCHS/GCE sensor demonstrated outstanding selectivity, reproducibility, and stability. The recovery percentages of AA, DA, and UA in real samples, including a vitamin C tablet, DA injection, and human urine, range from 99.8–110.9%, 99.04–100.45%, and 98.80–100.49%, respectively. Overall, the proposed sensor can serve as a useful reference for the construction of a high-performance electrochemical sensing platform.

## 1. Introduction

Ascorbic acid (AA), dopamine (DA), and uric acid (UA) are small bioactive molecules that are widely distributed in human blood, urine, and the central nervous system, playing a crucial role in human metabolism and the circulatory system [1,2,3]. AA, as an antioxidant, participates in various biological reactions and is used as a therapeutic agent for scurvy, acquired immune deficiency syndrome (AIDS), and cancers [4,5,6]. DA is an excitatory neurotransmitter, regulating endocrine as well as mental and physical activities. A lack of DA can cause heart disease, Parkinson’s disease, and other neurological diseases in humans [7,8]. UA is the primary end product of the metabolic breakdown of purine nucleotides, and it is one of the most important antioxidants in human fluids, such as urine and serum. Excessive UA in the human body can cause several diseases, such as gout, hypertension, arthritis, and hyperuricemia [9,10,11]. Therefore, there is an urgent need to develop a rapid, sensitive, and reliable method for the simultaneous detection of AA, DA and UA, considering their concurrent presence in physiological processes [12,13,14,15].

Until now, numerous analytical techniques have been applied for the detection of AA, DA, and UA, such as fluorescence spectroscopy [16,17], colorimetry [18,19], capillary electrophoresis [20,21], and high-performance liquid chromatography [22]. Despite achieving excellent reproducibility, wide detection range, and high accuracy, these conventional methods require complex separation processes, a high level of professionalism, and time-consuming procedures, limiting their broader application. By contrast, the electrochemical methods offer numerous advantages, including simple operation, rapid response, high sensitivity, low cost, and high feasibility, demonstrating their potential in the rapid detection of trace target molecules [23,24,25]. However, when AA, DA, and UA coexist in a sample, their oxidation peak potentials usually overlap when using bare glassy carbon electrode (GCE), so it is difficult to detect them simultaneously. To address this issue, novel electrode materials have been developed by modifying GCE for the distinguishing of these three analytes, such as metal oxide/carbon composite material [26], nitrogen–carbon material [27], and carbon matrix composite material [28].

A mesoporous carbon hollow sphere (MCHS) is a three-dimensional carbon material with large cavities and porous carbon shells, exhibiting uniform geometry, tunable porosity, a large surface area, and small particle size [29,30]. Consequently, it has been widely applied in catalysis [31], supercapacitors [32], drug release [33], batteries [34], and energy storage [35]. Palladium (Pd) nanoparticles, as noble metal nanomaterials, have garnered considerable research interest owing to their excellent catalytic activity, cost-effectiveness, and abundant availability. Nonetheless, the small size of Pd nanoparticles increases their surface free energy, causing their aggregation into larger nanoparticles. This leads to a significant decline in both the catalytic activity and stability of the resultant nanocomposite [36,37]. In the structure, metallic Pd nanoparticles are dispersed in the network of an MCHS, which not only affords a boost to conductivity but also greatly alleviates the aggregation of active sites. Simultaneously, the unique hollow structure facilitates the efficient mass transport and shortens the transmission path of electrons in the nanocomposite. Notably, nitrogen atoms within the MCHSs expose additional active sites that coordinate with Pd nanoparticles, forming robust Pd–N bonds [38]. This ensures the tight anchoring of Pd nanoparticles, endowing the nanocomposite with exceptional stability and electrochemical performance [39,40]. This feature can be helpful in the detection of AA, DA, and UA. However, to the best of our knowledge, the modification of GCE with a Pd-doped MCHS (Pd/MCHS) to realize the simultaneous determination of the three analytes has not yet been reported.

In this study, we applied a GCE modified with a Pd/MCHS nanocomposite for the concurrent detection of AA, DA, and UA. The distinctive hollow cavity structure of an MCHS not only facilitated the uniform dispersion of Pd nanoparticles but also prevented their aggregation during the fabrication process. The prepared Pd/MCHS/GCE sensor exhibited excellent sensitivity and selectivity in the individual detection of each analyte as well as in the simultaneous detection of the triad. According to the above considerations, the electrochemical sensor based on the Pd/MCHS nanocomposite proposed in this paper provides an efficient and sensitive method for the detection of biological small molecules, broadens the application range of the Pd/MCHS nanocomposite, and enriches the sensing mechanism in the field of biological sensors.

## 2. Results and Discussion

### 2.1. Characterization of Pd/MCHS

**Figure 1:**
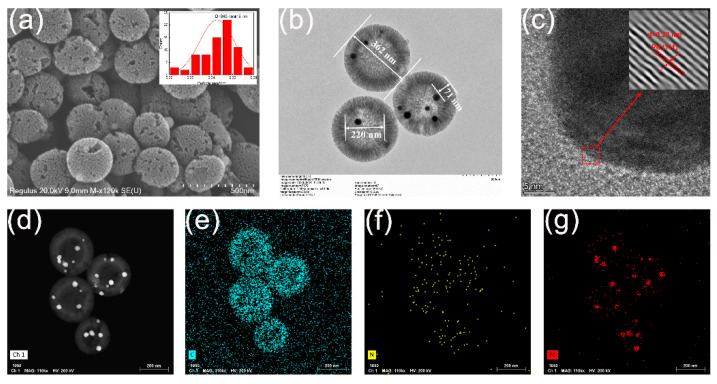
(**a**) SEM (inset: size distribution), (**b**) TEM, (**c**) HRTEM, and (**d**–**g**) elemental mapping images of Pd/MCHS nanocomposite.

SEM and TEM were used to reveal the microscopic morphologies of the Pd/MCHS/GCE and Pd/MCHS nanocomposites. The SEM image in Figure 1a shows that the Pd/MCHS/GCE has regular spherical morphology with a rough surface. The electrode surface appears as a broken ball, indicating that the nanocomposite has a hollow mesoporous structure with a size of approximately 345 nm, which contains uniformly dispersed Pd nanoparticles [41]. This arrangement significantly enhances the surface area of the Pd/MCHS material. According to the TEM image in Figure 1b, the Pd/MCHS nanocomposite exhibits a uniform spherical structure, where the diameter of spheres is approximately 362 nm, the diameter of the hollow structure is approximately 220 nm, and the thickness of the porous carbon shell is approximately 71 nm. Moreover, the Pd nanoparticles are evenly distributed within the MCHS material, avoiding particle aggregation, which highlights the outstanding anchoring capability of an MCHS. Notably, the hollow carbon nanosphere structure of the Pd/MCHS nanocomposite provides additional active sites through radical channels, facilitating electrode/electrolyte contact and enhancing the electrochemical performance of the prepared sensor [35].

In the high-resolution TEM (HRTEM) image of the Pd/MCHS nanocomposite (Figure 1c), an obvious interplanar spacing of 0.23 nm, corresponding to Pd (111), is observed for the ultrafine Pd nanoparticles. This indicates a face-centered cubic (fcc) structure of the Pd crystal lattice [29,42]. The elemental distribution of the Pd/MCHS nanocomposite was examined through EDS. The element mapping images in Figure 1d–g verify the homogeneous distribution of C, O, N, and Pd across the Pd/MCHS framework. Remarkably, the Pd nanoparticles are uniformly dispersed in the Pd/MCHS nanocomposite without any aggregation. These findings validate the successful synthesis of finely dispersed Pd nanoparticles firmly anchored onto the electrocatalytic substrate.

**Figure 2:**
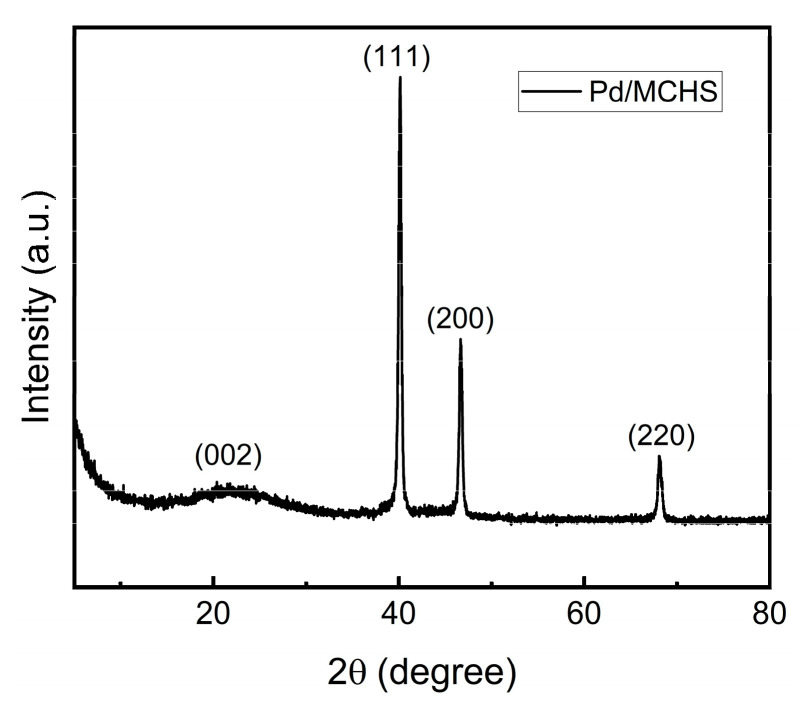
XRD pattern of Pd/MCHS nanocomposite.

The crystal structure of the Pd/MCHS nanocomposite was examined by XRD, and the results are shown in Figure 2. The sample exhibits a distinct diffraction peak at 24.6°, which is attributed to the (002) facet of graphitic carbon, confirming the presence of an amorphous structure. Furthermore, the fcc structure of the Pd crystal is confirmed by the diffraction peaks at 40.0°, 46.0°, and 68.0°, corresponding to the (111), (200), and (220) facets, respectively [43].

**Figure 3:**
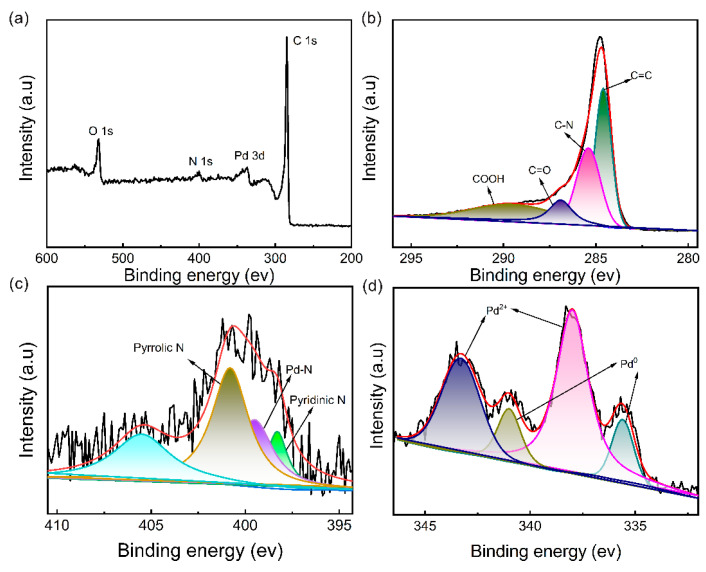
(**a**) XPS survey spectra of Pd/MCHS nanocomposite and high-resolution spectra of (**b**) C 1s, (**c**) N 1s, and (**d**) Pd 2p.

XPS was employed to further verify the successful fabrication of the Pd/MCHS nanocomposite. The peaks corresponding to C, N, and Pd are evident in the XPS spectrum of nanocomposite (Figure 3a). The results were consistent with the EDS elemental mapping. The high-resolution C 1s spectrum shows distinct peaks corresponding to different carbon bonds: sp^2^ C=C at 284.6 eV, C-N at 285.4 eV, C=O at 286.9 eV, and COOH at 289.5 eV (Figure 3b). Furthermore, the N 1s spectra exhibits discernible peaks representing pyridinic N (397.58 eV), Pd–N (399.5 eV), pyrrolic N (400.54 eV), and oxidized nitrogen species (405.3 eV) (Figure 3c). Notably, the interaction between pyridine N and Pd nanoparticles (Pd–N) is strong, which is ascribed to the more negative charges and diffuse orbitals associated with pyridine N. This interaction can effectively prevent the agglomeration of Pd nanoparticles, thereby enhancing the electrocatalytic activity and stability of the Pd/MCHS nanocomposite. The Pd 2p spectrum reveals a pair of peaks at 335.6 and 341.0 eV, which correspond to the metallic Pd (PdO) in the Pd/MCHS nanocomposite. Additionally, the peaks at 338.0 and 343.3 eV are attributed to the Pd–N interaction or PdO species (Figure 3d) [39].

### 2.2. Electrochemical Behavior of the Pd/MCHS/GCE Sensor

**Figure 4:**
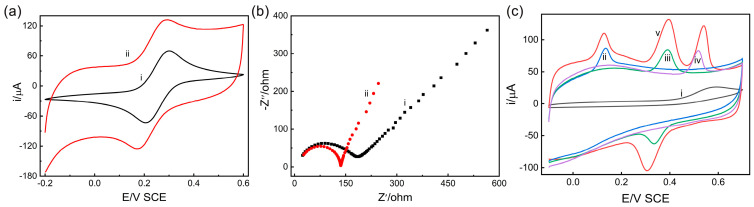
CV (**a**) and EIS (**b**) curves of GCE and Pd/MCHS GCE sensor in 0.1 M of KCl solution containing 5 mM [Fe(CN)_6_]^3−/4−^: (i) bare GCE; (ii) Pd/MCHS/GCE sensor. (**c**) CV curves of 1000 µM AA (ii), 40 µM DA (iii), 80 µM UA (iv), and mixed solution of 1000 µM AA, 40 µM DA, 80 µM UA in 0.1 M PBS (pH 3.0) at GCE (i) and Pd/MCHS/GCE sensor (v).

CV was employed to assess the interfacial charge transfer properties of GCE and the Pd/MCHS/GCE sensor. As illustrated in Figure 4a, distinct redox peaks are evident in the CV curve of unmodified GCE, which indicates the quasi-reversible electrochemical response of Fe(CN)_6_^3−/4−^. The modification of the GCE surface with Pd/MCHS results in a significant increase in the redox peak current. This suggested that the Pd/MCHS/GCE sensor had a large electrochemical active area, which can be ascribed to the synergistic effect of MCHS and Pd nanoparticles in promoting the electron transfer ability.

EIS is an effective method for evaluating the electrochemical features of a sensor by monitoring the changes in the charge transfer resistance (Rct) on the electrode surface. As shown in Figure 4b, an obvious semicircle is observed in the high frequency region of GCE. After the modification with the Pd/MCHS material, the semicircle diameter notably decreases. This reduction can be attributed to the enhanced electrical conductivity of the Pd/MCHS nanocomposite, facilitating the transfer of electrons in the redox reaction. The EIS curves are in agreement with the CV results, further verifying the successful assembly of the proposed electrochemical sensor.

**Figure 5:**
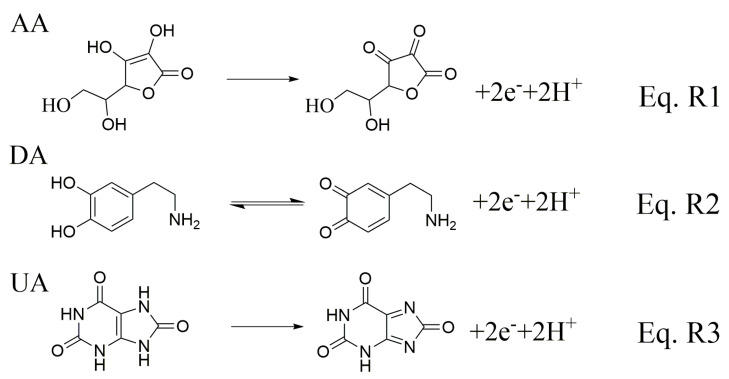
Electrochemical response of AA, DA, and UA on the Pd/MCHS/GCE sensor.

The individual electrochemical behaviors of AA, DA, and UA on the Pd/MCHS/GCE sensor were investigated by CV (Figure 4c). In the mixed solution of AA (1000 µM), DA (40 µM), and UA (80 µM), the oxidation peaks are located at 0.126, 0.396, and 0.54 V, respectively. Under an AA concentration of 1000 µM, the observed oxidation peak was chemically irreversible, which was attributed to the transformation of the hydroxyl group into a carbonyl group in the furan ring of AA. This oxidation pathway of AA can be expressed by the following equation R1 (Figure 5). Similarly, in 0.1 M PBS solution containing 40 µM DA, a distinctive pair of redox peaks is observed, indicative of the two-electron oxidation of DA to dopamine quinone, which is subsequently reduced back to DA. This redox transformation was shown in equations R2. In addition, in a 0.1 M PBS solution containing 80 µM UA, well-defined oxidation peaks are observed. The CV curve elucidates that UA is oxidized to a quinonoid form, and the corresponding oxidation pathway was presented in equation R3 [44,45]. Thus, the Pd/MCHS/GCE sensor demonstrates excellent ability for the simultaneous determination of the three analytes of interest.

**Figure 6:**
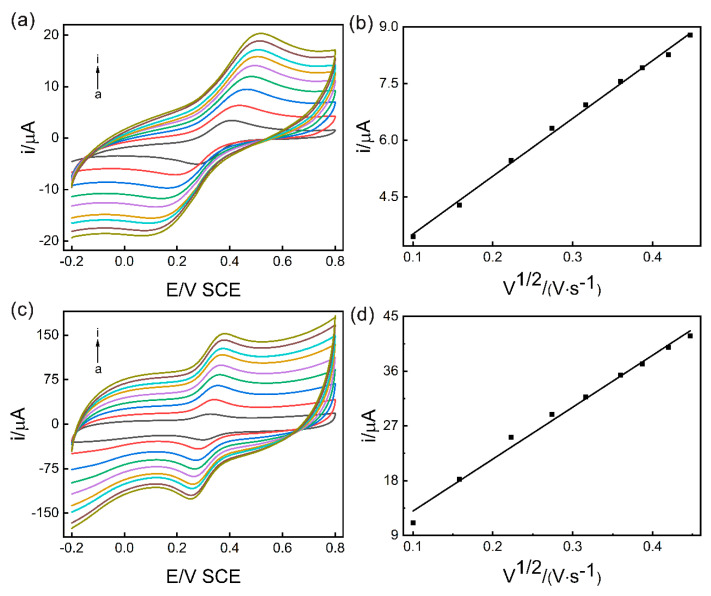
CV curves of GCE (**a**) and Pd/MCHS/GCE sensor (**c**) in 0.1 M of KCl solution containing 1 mM [Fe(CN)_6_]^3−/4−^. Linear relationship between the peak current (Ip) and the square root of the scan rate of GCE (**b**) and Pd/MCHS/GCE sensor (**d**).

The dynamic surface area of GCE and the Pd/MCHS/GCE sensor was calculated by using Randles–Sevcik equation (I_p_ = (2.69 × 10^5^) n^3/2^A D_0_^1/2^v^1/2^c_0_^∗^) (where n is the number of electrons involved in the reaction, i.e., equal to 1, A is effective area of electrode, D_0_ is diffusion coefficient (for K_3_[Fe(CN)_6_] is 7.6 × 10^−6^ cm^2^/s, v is scan rate and c_0_^∗^ is concentration of K_3_[Fe(CN)_6_] (1.0 mM)). From the slope of the plot seen in Figure 6, I_p_ = f (v^1/2^), the effective surface area of GCE and the Pd/MCHS/GCE sensor were found to be 0.0206 and 0.115 cm^2^. The effective surface area of the proposed sensor was obviously increased, which is conducive to the enrichment of target molecules on the sensor, the occurrence of electrochemical reactions, and the sensitive detection of small biological molecules [46,47,48].

The recognition mechanism of the Pd/MCHS/GCE sensor for the three target molecules is described as follows. The Pd/MCHS nanocomposite can provide abundant surface active sites, increase the collision probability between materials and target molecules, and finally enhance the detection signal of target molecules. Furthermore, the N doping in the Pd/MCHS nanocomposite improves the catalytic activity and biocompatibility of the materials, so that the electrons can be efficiently transferred between the Pd/MCHS/GCE sensor and the target molecules, which is conducive to the sensitive detection of AA, DA, and UA. The Pd/MCHS nanocomposite provides a favorable microenvironment to the target analyte. A small amount of AA, DA, and UA can trigger the composite to release a large number of signal molecules, resulting in a strong response signal, thereby achieving the rapid detection of AA, DA, and UA.

### 2.3. Optimization of Experimental Conditions

#### 2.3.1. Effect of Different Buffer Solutions

**Figure 7:**
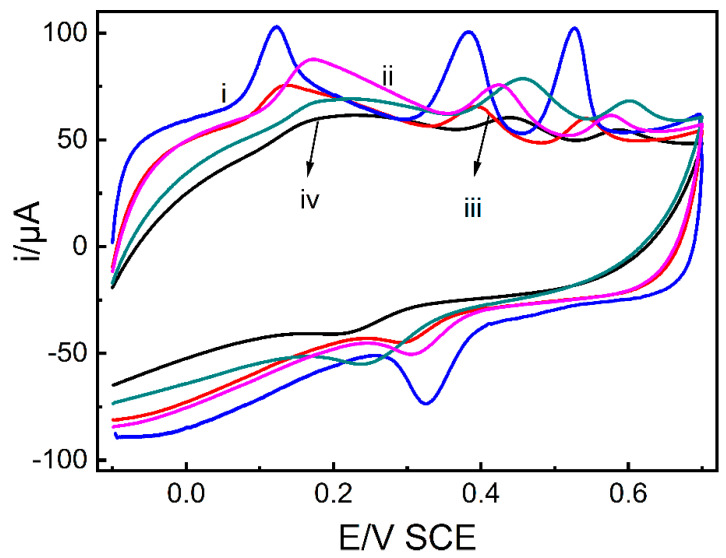
Effect of supporting electrolyte type on the sensor response: (i) PBS, (ii) citric acid-sodium citrate buffer solution, (iii) Britton–Robinson (B–R) solution, (iv) ABS, and (v) glycine-hydrochloric acid buffer solution.

The electrochemical behavior of AA, DA and UA on the Pd/MCHS/GCE sensor depends on various factors, such as the type of supporting buffer solution, pH value, the amount of Pd/MCHS nanocomposite, and scan rate. To obtain the optimal supporting buffer solution, the CV method was utilized to examine the oxidation behavior of AA, DA, and UA in PBS (i), citric acid-sodium citrate buffer solution (ii), Britton–Robinson (B–R) solution (iii), acetic acid sodium acetate buffer solution (ABS) (iv), and glycine-hydrochloric acid buffer solution (v), and the results are shown in Figure 7. Compared with the other four buffer solutions, PBS exhibited the highest electrochemical response in the detection of AA, DA and UA, and the corresponding peak currents are 36.24, 44.08, and 48.43 µA, respectively. Therefore, PBS was chosen as the buffer solution in the subsequent measurements.

#### 2.3.2. Effect of pH

**Figure 8:**
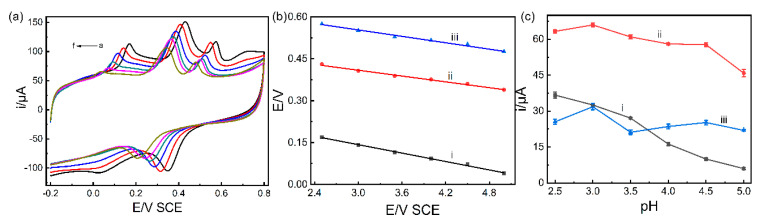
(**a**) CV curves for the oxidation of AA (1000 μM), DA (40 μM), and UA (80 μM) on the Pd/MCHS/GCE sensor under various pH values ranging from 2.5 to 5 (a–f); (**b**) influence of pH on the oxidation peak potentials; (**c**) influence of pH on the oxidation peak current: (i) AA; (ii) DA, and (iii) UA.

The impact of buffer pH on the current response of the Pd/MCHS/GCE sensor for the determination of 1000 μM AA, 40 μM DA, and 80 μM UA, was systematically investigated over a range of pH values from 2.5 to 5, and the results are shown in Figure 8a. It can be seen in Figure 8b that the peak potential (Ep) exhibited a discernible negative shift as the pH of the electrolyte increases. This indicated a concurrent electron and proton transfer during the electrochemical oxidation reaction [49,50]. The relationship between Ep and pH can be expressed as follows: Ep (V) = 0.0503 pH + 0.294 (R^2^ = 0.995) for AA, Ep (V) = 0.0347 pH + 0.513 (R^2^ = 0.995) for DA, and Ep (V) = 0.0383 pH + 0.668 (R^2^ = 0.993) for UA. Figure 8c further illustrates that the peak currents of these analytes depend on the pH value of the buffer solution. Consequently, 0.1 M PBS at pH 3.0 was selected as the supporting electrolyte solution for this experiment.

#### 2.3.3. Effect of the Amount of Pd/MCHS

**Figure 9:**
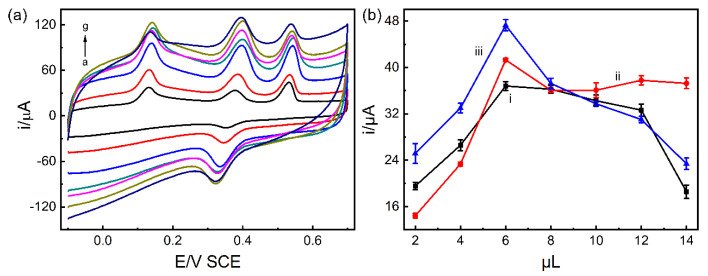
(**a**) CV curves for the oxidation of AA (1000 μM), DA (40 μM), and UA (80 μM) on the Pd/MCHS/GCE sensor under different amounts of modifier ranging from 2 µL to 14 µL (a–g). (**b**) Effect of the amount of Pd/MCHS dispersion on the oxidation peak current: (i) AA, (ii) DA, and (iii) UA.

The optimal loading amount of Pd/MCHS material on the GCE electrode was investigated through seven control experiments using different amounts of Pd/MCHS (2, 4, 6, 8, 10, 12, and 14 µL). It can be seen in Figure 9 that, as the amount of Pd/MCHS increases, the peak current of AA, DA, and UA grows dramatically and reaches its maximum value at 6 µL. Then, the current response rapidly decreases with a further increase in the amount of Pd/MCHS. This may be because the elevated amount of Pd/MCHS leads to its aggregation, which hinders the interfacial transport of electrons. These results suggested that 6 µL was the optimal amount of Pd/MCHS for the oxidation of AA, DA, and UA on the Pd/MCHS/GCE sensor.

#### 2.3.4. Effect of Scan Rate

**Figure 10:**
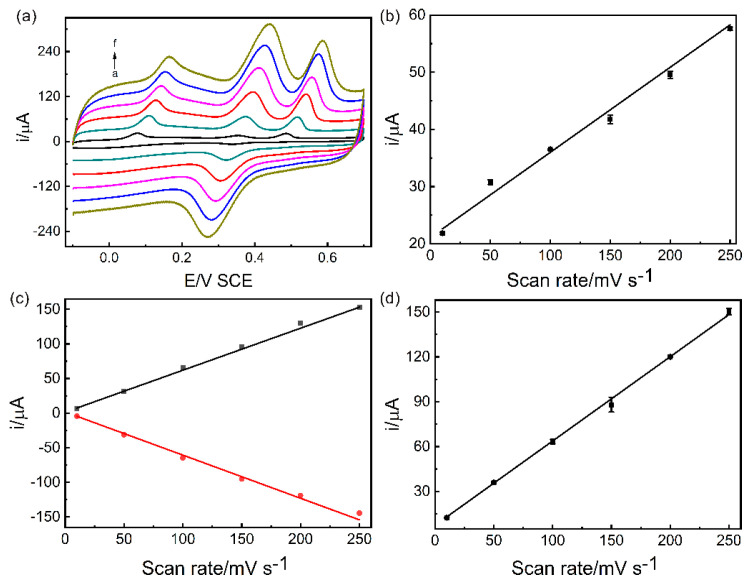
(**a**) CV curves of Pd/MCHS/GCE sensor in PBS (pH = 3.0, 0.1 M) containing 1000 μM AA, 40 μM DA, and 80 μM UA under scan rates from 10 to 250 mV·s^−1^ (a–f). A linear relationship is observed between the oxidation peak current and scan rates: (**b**) AA, (**c**) DA, and (**d**) UA.

Figure 10a shows the electrochemical response of the Pd/MCHS/GCE sensor in 0.1 M PBS (pH 3.0) solution containing 1000 μM AA, 40 μM DA, and 80 μM UA at different scan rates. As the scan rate increases from 10 to 250 mV·s^−1^, both oxidation and reduction peak currents increase linearly. Figure 10b–d displays the linear relationship between the peak current (I) and scan rate (v), which can be described using the following regression equations: I_AA_ = 0.283 C_AA_ + 15.923 (R^2^ = 0.999), I_DA_ = 0.612 C_DA_ + 0.011 (R^2^ = 0.999), and I_UA_ = 0.506 C_UA_ + 3.541 (R^2^ = 0.999). These findings indicated that the electrochemical oxidation process of the Pd/MCHS/GCE sensor toward AA, DA, and UA follows a surface-controlled mechanism [51].

The above experiments were used to determine the optimal conditions for the application of the Pd/MCHS/GCE sensor in the electrochemical detection of AA, DA, and UA.

#### 2.3.5. Selection of Analytical Method

**Figure 11:**
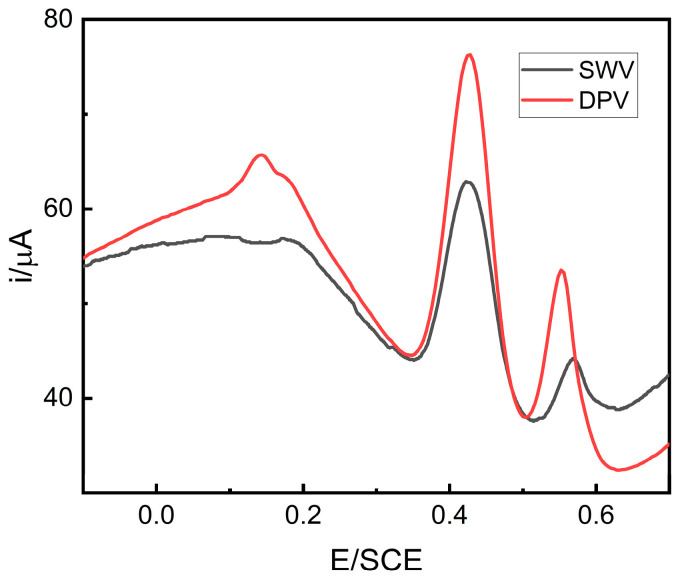
Selection of an analytical method for the detection of AA, DA, and UA.

In order to select the best technique method, square wave voltammetry (SWV) and differential pulse voltammetry (DPV) were used to investigate the effects of the Pd/MCHS/GCE sensor with an AA, DA and UA mixed solution, and the obtained signals are shown in Figure 11. The working parameters of SWV were as follows: the amplitude was 25 mV, the frequency was 15 Hz; the DPV had an amplitude of 50 mV and a pulse width of 200 mV. Because the three compounds exhibited higher peak currents and better peak shape in when separated further and further, DPV was used to analyze AA, DA, and UA in the following experiments.

### 2.4. Individual and Simultaneous Determination of AA, DA, and UA

**Figure 12:**
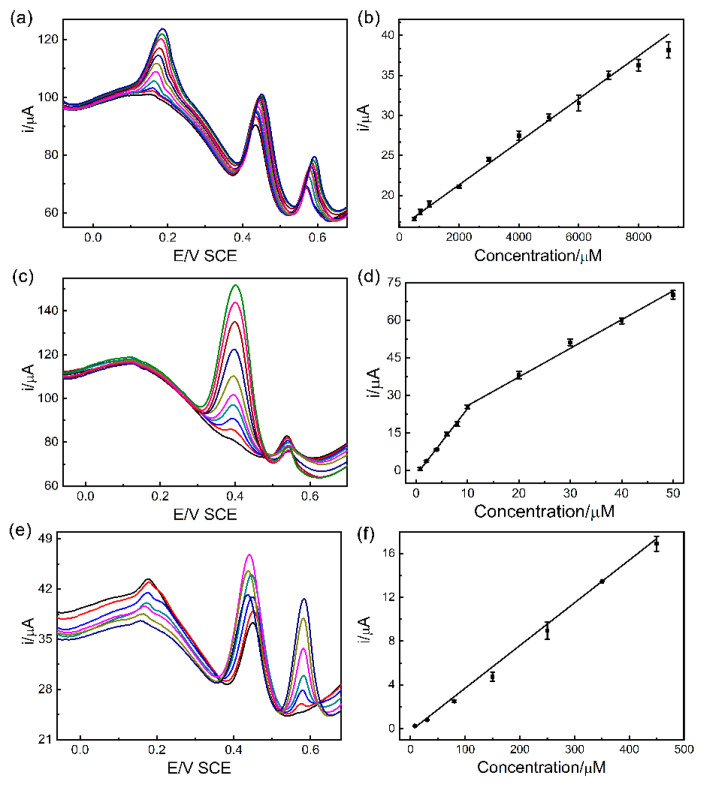
DPV curves of Pd/MCHS/GCE sensor in PBS (pH = 3.0, 0.1 M) solution containing (**a**) 10 µM DA, 200 µM UA, and different con-centrations of AA from 500 to 9000 µM; (**c**) 1000 µM AA, 200 µM UA, and different concentrations of DA from 0.5–10 µM to 10–50 µM; (**e**) 1000 µM AA, 10 µM DA, and different concentrations of UA from 8 to 450 µM. A linear relationship is observed between Ip and the corresponding concentration (C) of (**b**) AA, (**d**) DA, and (**f**) UA.

DPV with high sensitivity and resolution was employed for the determination of AA, DA, and UA under optimal conditions. As depicted in Figure 12a,c,e, when keeping the concentrations of the two analytes constant while increasing the concentration of the third analyte, a consistent variation in the peak current value of the third analyte was observed. As seen in Figure 12b,d,f, the linear relationship between the peak current value (Ip) and concentration (C) can be described by the following equations: Ip (µA) = 0.00269 C_AA_ (µM) + 15.940, Ip (µA) = 2.644 C_DA_ (µM) − 1.757, Ip (µA) = 1.145 C_DA_ (µM) + 14.436, and Ip (µA) = −0.198 C_UA_ (µM) + 0.039. The limit of detection (LOD) values were calculated to be 31.10, 0.036, and 5.00 µM, respectively.

When varying the AA concentration and keeping the DA and UA concentrations constant, AA is more likely to form a stable adsorption layer in the Pd/MCHS/GCE sensor. DA and UA will compete with AA for the adsorption site on the sensor, and continue to be crowded out, affecting their mass transmission and diffusion behavior, which resulted in its peak current increasing with the change of concentration. The same current changes occurred when DA and UA concentrations change.

**Figure 13:**
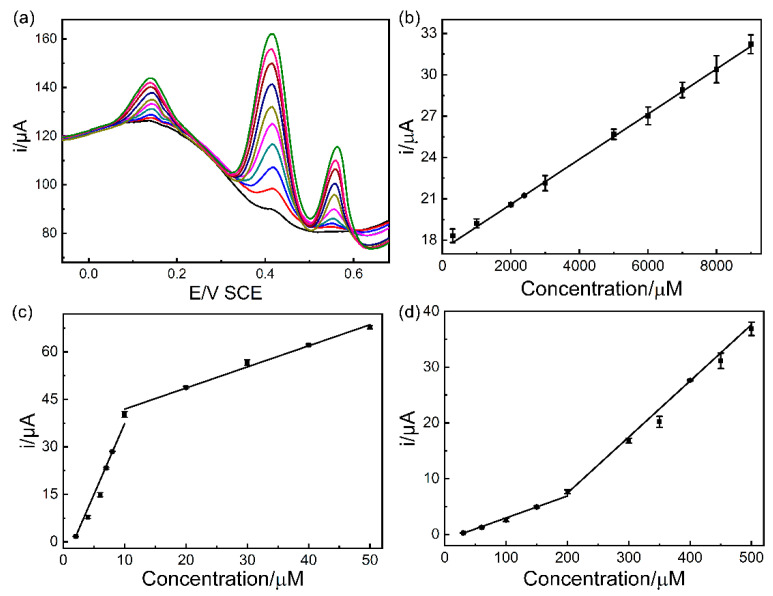
(**a**) DPV curves of Pd/MCHS/GCE sensor in PBS (pH = 3.0, 0.1 M) solution containing 300–9000 µM AA, 2–50 µM DA, and 20–500 µM UA. A linear relationship is observed between the peak current (Ip) and concentration (C) of (**b**) AA, (**c**) DA, and (**d**) UA.

Furthermore, the concurrent detection of AA, DA and UA with different concentrations was thoroughly examined under the optimal experimental conditions. Figure 13 shows the variation in the current response as a function of the concentrations of AA, DA, and UA. The linear concentration ranges for AA, DA, and UA were determined to be 300–9000 µM, 2–50 µM, and 20–500 µM, with LOD values of 51.00, 0.14, and 4.96 µM, respectively. These findings demonstrated that the Pd/MCHS/GCE sensor can accurately determine AA, DA, and UA.

A comparative analysis with other sensors revealed that the Pd/MCHS/GCE sensor exhibited a desirable linear range for the measurement of AA, DA, and UA (Table 1). Notably, the proposed sensor demonstrated a relatively lower LOD, which is attributed to the abundant surface active sites, excellent electrical conductivity, and suitable N doping in the Pd/MCHS nanocomposite. Overall, with a straightforward electrochemical assembly strategy, a novel sensing platform with a Pd/MCHS modified electrode can be constructed, which displayed remarkable performance for the detection of AA, DA, and UA.

### 2.5. Reproducibility, Stability, and Anti-Interference Performance of Pd/MCHS/GCE Sensor

**Figure 14:**
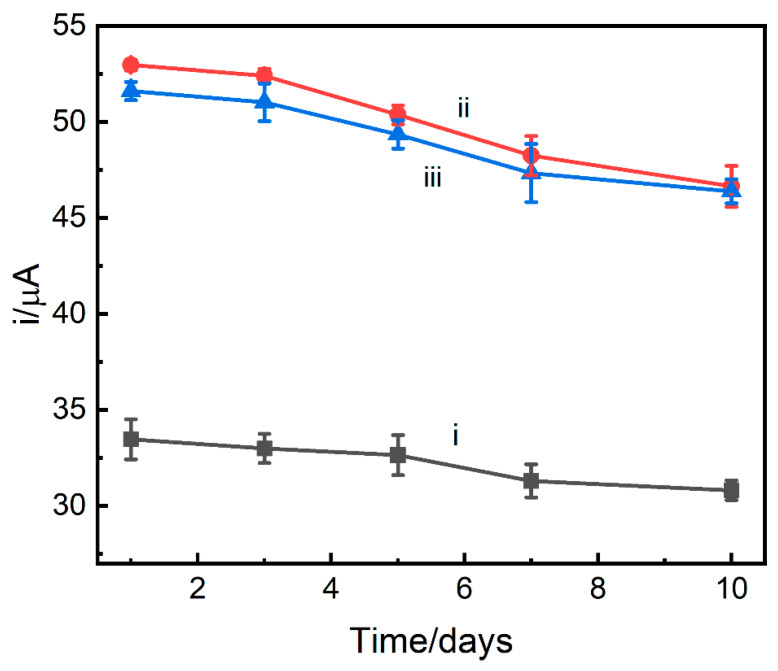
Stability of Pd/MCHS/GCE sensor toward 1000 μM AA (i), 40 μM DA (ii), and 80 μM UA (iii) for 10 days.

The reproducibility and stability of the Pd/MCHS/GCE sensor were investigated in a 0.1 M PBS solution with a pH of 3.0, containing 1000 µM AA, 40 µM DA, and 80 µM UA using DPV technology under the optimal conditions. The reproducibility of the sensor was evaluated by fabricating six identical modified electrodes for the mixed solution, ensuring consistency in the manufacturing process. As shown in Table 2, the average relative standard deviation (RSD) for AA, DA, and UA are 4.60%, 1.96%, and 2.82%, respectively, signifying the excellent reproducibility of the Pd/MCHS/GCE sensor. Furthermore, the fabricated sensor was maintained at 4 °C for 10 days to evaluate its stability, and the current response of the electrode was randomly monitored at 1, 3, 5, 7, and 10 days. As shown in Figure 14, after storage for 10 days, the peak current density of AA, DA, and UA decreased by only 12.64%, 12.40%, and 10.83%, respectively, verifying the excellent stability of the Pd/MCHS/GCE sensor.

**Figure 15:**
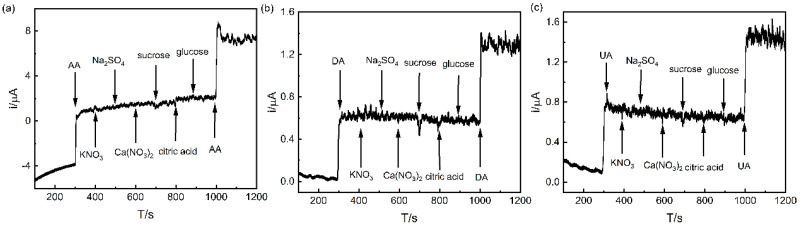
Selectivity of the Pd/MCHS/GCE sensor toward AA (**a**), DA (**b**), and UA (**c**) in the presence of different interfering species including KNO_3_, Na_2_SO_4_, Ca(NO_3_)_2_, sucrose, citric acid, and glucose.

To confirm the high selectivity of the Pd/MCHS/GCE sensor for the determination of AA, DA, and UA, the recognition performance of the sensor for different substances (KNO_3_, Na_2_SO_4_, Ca(NO_3_)_2_, sucrose, citric acid, and glucose) was investigated under a voltage of 0.1 V (AA), 0.4 V (DA), and 0.55 V (UA), and the results were shown in Figure 15. Compared to the current signal obtained from AA (1000 µM), DA (30 µM), and UA (30 µM), the interfering species (2000 µM) do not induce an obvious current response. Due to the attractive electronic properties and high catalytic activity of the Pd/MCHS nanocomposite, it can provide electron tunneling, enabling electrons to transfer between the active site of the nanocomposite and the Pd/MCHS/GCE sensor, and can selectively improve the mass diffusion of the target molecule, so that the constructed electrochemical sensor shows a highly sensitive and selective electrochemical sensing performance in the detection of AA, DA and UA.

**Figure 16:**
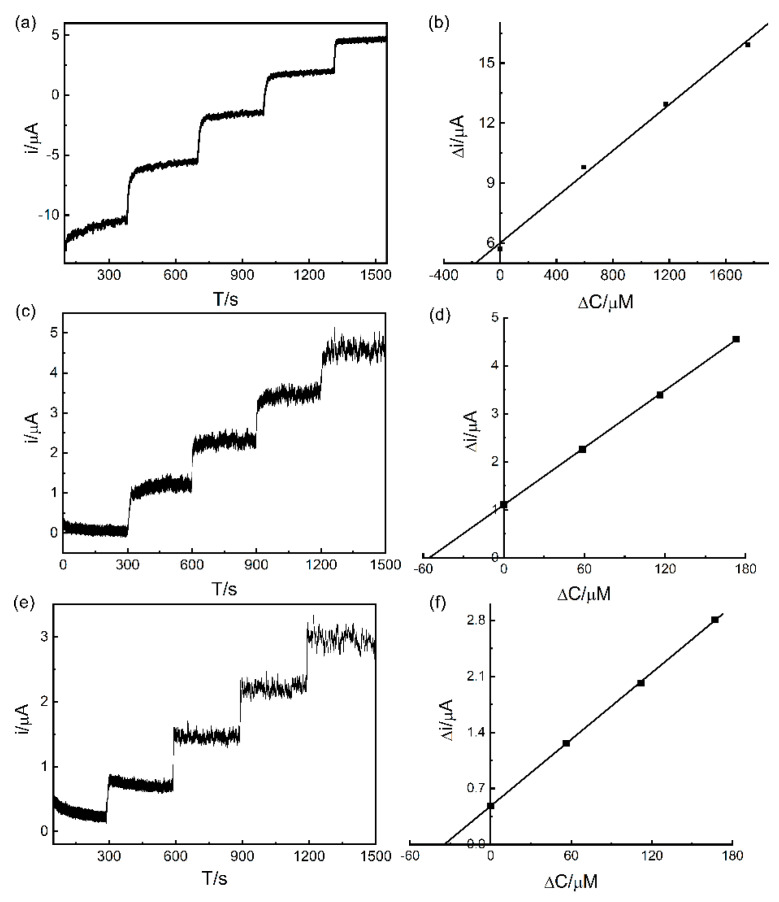
(**a**,**c**,**e**) Amperometric response of the sensor under sequential addition of vitamin C tablet, DA injection, and human urine samples, with the AA, DA and UA standard solutions being added three times. (**b**,**d**,**f**) A linear relationship is observed between ΔI and ΔC for vitamin C tablet, DA injection, and human urine samples on the Pd/MCHS/GCE sensor in PBS solution.

The amperometry method is an electrochemical method for the quantitative analysis of electrochemically active substances by applying a constant potential to the study electrode. Because this method can maintain constant potential and reduce the influence of the double layer charging current, it is often used to detect real samples. Therefore, it was used to detect AA, DA, and UA in a vitamin C tablet, DA injection, and human urine to assess the practical feasibility of the Pd/MCHS/GCE sensor. The specific parameters were set as follows: the initial potential was 0.55 V, the sampling interval was 0.1 s, and the sampling time was 1600 s. As shown in Figure 16, the recoveries were calculated based on the relationship between ΔC and ΔI. The average recoveries of AA, DA, and UA in the Pd/MCHS/GCE sensor were 99.8–110.9%, 99.04–100.45%, and 98.80–100.49%, respectively.

In order to verify the reliability of the electrochemical sensor method, the data of real samples detected using a UV–Vis spectrophotometer were used for comparison. The maximum absorption wavelengths of ascorbic acid and uric acid were 280 nm and 400 nm, respectively. By drawing standard curves and adopting a standard addition method, the recoveries of ascorbic acid and uric acid were in the range of 98.10–100.25% and 100.36–101.82% (Table 3), respectively, which was consistent with the data of real samples detected using the electrochemical sensor method, indicating that the electrochemical detection of AA, DA and UA contents in real samples had strong feasibility.

## 3. Experimental Section

### 3.1. Chemicals and Materials

AA, DA, UA, citric acid, calcium nitrate, sucrose, glucose, SiO_2_, Na_2_PdCl_4_, sodium hydroxide (NaOH), resorcol, formaldehyde, and tetraethyl orthosilicate (TEOS) were purchased from Shanghai Macklin Biochemical Co., Ltd. Sodium hydrogen phosphate (Na_2_HPO_4_), sodium dihydrogen phosphate (NaH_2_PO_4_), phosphoric acid (H_3_PO_4_), ammonia (NH_3_·H_2_O), anhydrous alcohol (C_2_H_6_O), potassium nitrate (KNO_3_), and sodium sulfate (Na_2_SO_4_) were procured from Tianjin Deen Chemical reagent Co., Ltd., Tianjin, China. Al_2_O_3_ power and all electrodes were purchased from Shanghai Xianren Instrument. All the chemicals and reagents are of analytical grade and used as received without further purification. Ultrapure water with a resistivity of 18.2 MΩ cm was used throughout the experiment.

### 3.2. Synthesis of Pd/MCHS Nanocomposite

Firstly, the Pd/MCHS nanocomposite was synthesized according to our previous report. Briefly, 400 mg SiO_2_ was dissolved into a solution of ethanol (70 mL), H_2_O (10 mL), and NH_3_·H_2_O (3 mL) under magnetic stirring for 30 min. Next, Na_2_PdCl_4_ (0.265 g) was added in the above solution and stirred for 1 h at 300 rpm. Subsequently, resorcol (0.4 g), formaldehyde (0.56 mL), and tetraethyl orthosilicate (TEOS) (3.46 mL) were added in the solution, and the reaction mixture was continuously stirred for 24 h. After the completion of the reaction, the mixture was consecutively washed with ethanol and ultrapure water to remove the impurities and by-products. Then, the resulting black precipitate was dried overnight at 60 °C under vacuum. The obtained precursor was heated to 800 °C for 2.5 h in an Ar atmosphere at a rate of 2 °C/min. The silica template was removed with NaOH (20%) to obtain the final product, denoted as Pd/MCHS [57].

### 3.3. Characterization and Electrochemical Methods

The morphology of the samples was examined using field emission scanning electron microscopy (FE-SEM; JSM-7610FPlus microscope, JEOL, Tokyo, Japan) and transmission electron microscopy (TEM; Talos F200i microscope, FEI, Hillsboro, OR, USA). The elemental composition and chemical states were investigated through X-ray photoelectron spectroscopy (XPS; Thermo Scientific ESCALAB 250Xi). The crystallographic information was obtained using X-ray diffraction (XRD; Rigaku D/Max-2400). The electrochemical measurements were performed using a CHI 660E electrochemical analyzer with a three-electrode system. A saturated calomel electrode (SCE) and a platinum wire were used as the reference and auxiliary electrodes, respectively, and either GCE or the modified Pd/MCHS/GCE was used as the working electrode. Cyclic voltammetry (CV) and differential pulse voltammetry (DPV) curves were recorded within the potential range of 0.2 to 0.8 V. The electrochemical impedance spectroscopy (EIS) tests were conducted in a 0.1 M KCl solution containing 5 mM Fe(CN)_6_^3−/4−^ in the frequency range of 0.01–100 Hz. Furthermore, the amperometric response (I-t) curves were generated to assess the anti-interference capability and conduct real sample analysis. These experiments were performed at constant potentials of 0.1 V (AA), 0.42 V (DA), and 0.55 V (UA) under magnetic stirring at 50 rpm/min.

### 3.4. Analysis of Real Samples

Vitamin C tablets and DA injection solution were procured from a local pharmacy, while the UA sample was obtained from a sophomore student at Henan University of Science and Technology. The samples were prepared as follows. Firstly, 0.1 g of the vitamin C tablet was ground and dissolved in 10 mL phosphate-buffered saline (PBS) solution to obtain the actual AA sample. The 20 mg/2 mL DA injection solution and the urine sample were each subjected to a tenfold dilution with 0.1 M PBS (pH = 3.0), without undergoing additional treatment. To obtain the content of AA, DA, and UA in the actual samples, the standard addition method was employed. The recovery percentages were calculated as follows: Recovery (%) = (found/added) × 100%. This approach ensured a rigorous quantitative assessment of the targeted analytes in the real-world samples.

### 3.5. Preparation of Modified Electrode

**Scheme 1:**
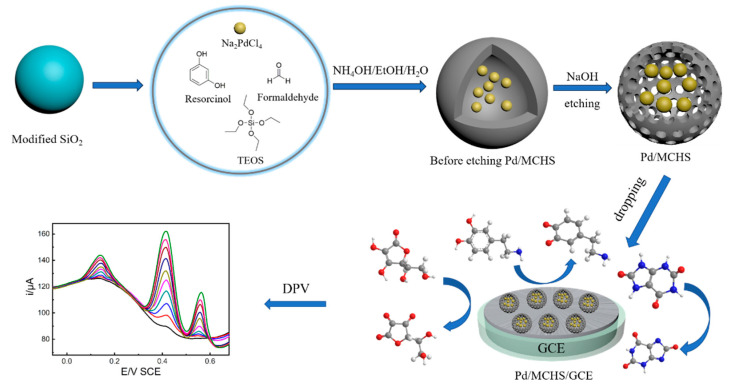
Schematic diagram of the preparation of Pd/MCHS/GCE sensor for the detection of AA, DA, and UA.

Firstly, the bare GCE was polished with 1 µm and 0.05 µm Al_2_O_3_ powders, followed by ultrasonic cleaning with 0.5 M H_2_SO_4_ and anhydrous ethanol, respectively. Subsequently, 1 mg Pd/MCHS was sonicated in a solution containing 0.5 mL distilled water, 0.5 mL anhydrous ethanol, and 6 µL Nafion for 10 min to prepare a homogeneous suspension. Then, the homogeneous suspension (6 µL, 1 mg/mL) was dropped on the pretreated GCE and dried using an infrared lamp to prepare the Pd/MCHS/GCE sensor. The preparation process of the Pd/MCHS/GCE sensor as well as the simultaneous detection of AA, DA, and UA using the fabricated sensor were illustrated in Scheme 1.

## 4. Conclusions

Our study has demonstrated the remarkable properties of the Pd/MCHS/GCE sensor in the molecular recognition of AA, DA and UA, providing a promising avenue for future research. The synergistic combination of MCHS and Pd has contributed to the excellent sensing performance of the electrode sensor and provided a reference approach for the electrochemical detection of small biological molecules. In addition, the non-toxic, profitable and environmentally friendly process further increased the appeal of this new sensing platform. Future studies can focus on the stability and repeatability of these electrochemical sensors over long periods of time and under complex environmental conditions. In addition, we can expand the performance of the Pd/MCHS/GCE sensor in serum and clinical sample applications to provide data references for clinical applications.

## Figures and Tables

**Table 1:** Comparison between the electrochemical performance of the proposed and other reported electrode sensors for the detection of AA, DA, and UA.

Electrode	Linear Range (µM)			Limit of Detection (µM)			Refs.
	AA	DA	UA	AA	DA	UA	
MWCNT-PEDOT/GCE	100–2000	10–330	10–250	100	10	10	[28]
ERGO/GCE	500–2000	0.5–60	0.5–60	250	0.5	0.5	[52]
Bare GCE	0–190	0–160	0–100	7.66	11.34	5.63	[53]
SDS/CPE	-	10–196	60–654	-	7.71	35.88	[54]
GEF/CFE	73.52–2305.53	1.36–125.69	3.98–371.49	73.52	1.36	3.98	[55]
Au-RGO/GCE	240–1500	6.8–41	8.7–53	51	1.4	1.8	[56]
Pd/MCHS/GCE ^a^	500–9000	0.8–50	8–450	31.10	0.036	5.00	This work
Pd/MCHS/GCE ^b^	300–9000	2–50	20–50	51.00	0.14	4.96	This work

^a^ Individual analysis; ^b^ Simultaneous analysis.

**Table 2:** Oxidation currents of 1000 µM AA, 40 µM DA, and 80 µM UA with six independent sensors.

	i_1_ (μA)	i_2_ (μA)	i_3_ (μA)	i_4_ (μA)	i_5_ (μA)	i_6_ (μA)	RSD (%)
AA	30.84	32.73	33.00	34.72	34.54	34.67	4.60
DA	50.90	49.96	50.25	48.46	48.75	48.92	1.96
UA	52.14	51.89	51.23	49.95	49.58	48.53	2.82

**Table 3:** Results of recoveries of AA and UA in real samples using UV–Vis spectrophotometer.

Samples	Detected		Found		Recovery (%)	
	AA (μg)	UA (μM)	AA (μg)	UA (μM)	AA	DA
1	10	2.79	9.81 ± 0.23	2.80 ± 0.13	98.10	100.36
2	12	2.99	12.03 ± 0.35	3.02 ± 0.18	100.25	101.00
3	15	3.29	14.93 ± 0.44	3.35 ± 0.22	99.53	101.82
4	20	3.79	20.03 ± 0.52	3.82 ± 0.31	100.15	100.79

## Data Availability

Data cannot be shared for ethical/privacy reasons. The data underlying this article cannot be shared publicly due to the privacy of individuals that participated in the study. The data will be shared on reasonable request to the corresponding author. All data generated or analyzed during this study were included in this published article.
